# Association Between Vitamin D Status and Undernutrition Indices in Children: A Systematic Review and Meta-Analysis of Observational Studies

**DOI:** 10.3389/fped.2021.665749

**Published:** 2021-06-04

**Authors:** Chunhua Song, Hongzhi Sun, Ben Wang, Chunli Song, Hongying Lu

**Affiliations:** ^1^Department of Pediatrics, Dezhou People's Hospital, Dezhou, China; ^2^Department of Pediatrics, Laoling People's Hospital, Laoling, China; ^3^Department of Pediatrics, Xiajin County People's Hospital, Dezhou, China

**Keywords:** vitamin D, undernutrition, stunting, wasting, underweight, meta-analysis

## Abstract

**Introduction:** Undernutrition, defined as stunting, wasting, and underweight, still implicates millions of infants and children worldwide. Micronutrients have pivotal effects on growth rate. The outcomes of vitamin D deficiency on undernutrition indices have stayed controversial. The object of current study is to answer this question: is there any association between vitamin D status and undernutrition indices?

**Methods:** The international databases were used for a systematic search to identify relevant observational studies in English up to January 2021. A random-effect model was applied to combine the results of included essays.

**Results:** Among 3,400 citations, 7 observational studies (4 cohorts and 3 cross-sectional) were eligible to enter in meta-analysis. Analysis of the lowest 8,295 children indicated that low vs. high serum level of vitamin D is directly associated with a higher risk of wasting (Summary Risk Estimate: 1.30; 95% CI: 1.04, 1.62; *I*^2^ = 0%). However, there is no significant association between vitamin status and risk of stunting (Summary Risk Estimate: 1.10; 95% CI: 0.72, 1.70; *I*^2^ = 81.6%) and underweight (Summary Risk Estimate: 1.12; 95% CI: 0.81, 1.56; *I*^2^ = 49.2%).

**Conclusion:** When comparing low and high serum vitamin D concentration categories, there is an inverse link between vitamin D status and wasting, but no relationship with stunting as well as underweight. However, further prospective and trial studies are required to deepen our understanding of these associations.

## Introduction

Children undernutrition (including wasting, stunting, and underweight) is still a serious challenge and puts medical, social, and economic burden, particularly in developing countries. It is estimated that the number of stunted and wasted children are 144 (21.3%) and 47 (6.9%) million worldwide, respectively. Notably, the prevalence of undernutrition is roughly double in low and middle-income countries (1–3). The risk of morbidity and mortality is more significant in children with undernutrition so that the etiology of about 45% of children mortality could be attributed to undernutrition (1, 4). These children usually experience cognitive and motor impairments, growth retardation, and weaker school performance (4, 5). Widespread factors contribute to malnutrition, such as genetic agents, political position, low socioeconomic status, infections, and inadequate dietary intake (5, 6).

Several former studies established the role of some micronutrients such as vitamin A, zinc, iron, and vitamin D in growth rates (7, 8). Although vitamin D's role in the metabolism of calcium and phosphorous and, consequently, skeletal growth has been proven in previous studies (9, 10), recent research has found biological functions. For example, there is an interaction between vitamin D and growth hormone (9, 10). Vitamin D inadequacy as a common nutritional deficiency is rarely diagnosed (11). The increase of vitamin D deficiency has been a big problem for global public health (12, 13).

Several investigations have explored the association of vitamin D status with undernutrition indices in children but have announced incongruous results (14–20). For instance, a survey on undernutrition indices in a cohort of HIV-exposed, uninfected infants concluded that vitamin D deficiency might increase the chance of wasting, while it did not observe any association with stunting and underweight (20).

Due to no research has elucidated this controversy, this systematic review and meta-analysis of published observational papers were performed to summarize findings regarding the association between vitamin D status and risk of wasting, stunting, and underweight in children.

## Method

This meta-analysis was conducted on essays that evaluated the link between serum vitamin D status and undernutrition as stunting, wasting, and underweight, among infants and children. The serum level of 25(OH) vitamin D was considered as a biomarker. This review was reported based on the Preferred Reporting Items of Systematic Reviews and Meta-Analysis (PRISMA) statement guidelines (21).

### Search Strategy

The databases of PubMed and Scopus were searched to identify relevant articles published up to January 2021. The studies with an observational design that met inclusion criteria were enrolled to be analyzed. The search terms [Medical Subject Heading (MeSH) and non-MeSH terms] were the following items: Vitamin D OR Cholecalciferol OR ergocalciferol OR 25-hydroxyvitamin D OR 25(OH)D OR 1-alpha hydroxyvitamin D3 OR 1,25 dihydroxyvitamin D3 OR 1,25 dihydroxycholecalciferol OR 25 hydroxycholecalciferol OR 25 hydroxyvitamin D OR calcitriol in combination with stunting OR wasting OR underweight OR thinness OR leanness OR stunted growth. If there was missing data in included studies, we contacted the author by email. Google Scholar databases and references of included essays and relevant reviews were also checked to find any missed study.

### Inclusion and Exclusion Criteria

Studies that had an observational design (cohort, cross-sectional, or case-control studies), conducted on children aged under 12 years, and reported sufficient data [hazard ratio (HR), relative risk (RR), and odds ratio (OR) with corresponding 95% CIs] regarding the association of serum vitamin D status and risk of wasting, stunting, and underweight were included. Studies with the following features were excluded: (1) adult population; (2) dietary vitamin D intake; (3) animal studies; (4) no English essays; (5) review articles, commentary, editorial, or letter; (6) intervention studies; (7) studies that had subjects with chronic diseases such as cancer, cystic fibrosis, and other conditions; (8) reporting the correlation coefficient instead of estimate risk.

### Quality Assessment

The valid tool of the Newcastle–Ottawa Scale (NOS) was used to ascertain the risk of bias of included essays (22). The total score of NOS is in the range of 0–9. If one study gets a score of ≥6, it is considered as high quality. The process of study qualification was performed by two independent researchers. If there was any discrepancy, researchers made the final decision by consultation and consensus.

### Data Extraction and Abstraction

A pre-made form was used by two researchers independently to extract the required data. The following data were extracted: name of the first author, date of publication, study design, follow-up duration (in prospective cohort studies), sample size, age, gender, geographic situation, serum vitamin D assessment method, and outcome evaluating approach.

### Statistical Analysis

The random-effects model was used to calculate estimated risk with 95% CIs to compare the highest and lowest serum level of vitamin D. The inverse variance method was applied to compute the standard error (SE) value of the logarithmic OR/HR/RR for each essay, and the estimated variance of the logarithmic RR was used to assess the weight of each study. *I*^2^ test was applied to assess heterogeneity between included studies. The *I*^2^ values of 25–50%, 50–75%, and >75% were considered as low, moderate, and high between-study heterogeneity, respectively (23). Subgroup analysis was conducted to identify source of heterogeneity according to the following variables: study design, participant age range, number of cases, and serum vitamin D assessment tool. For evaluating publication bias, the funnel plot and egger test were used (24). To examine the effect of one by one of the included studies on overall results, a sensitivity analysis was applied. All statistical analyses were done using STATA software version 15.1 (Stata Corporation, College Station, Texas, USA). The *P*-value under 0.05 was presumed as significant.

## Results

**Figure 1:**
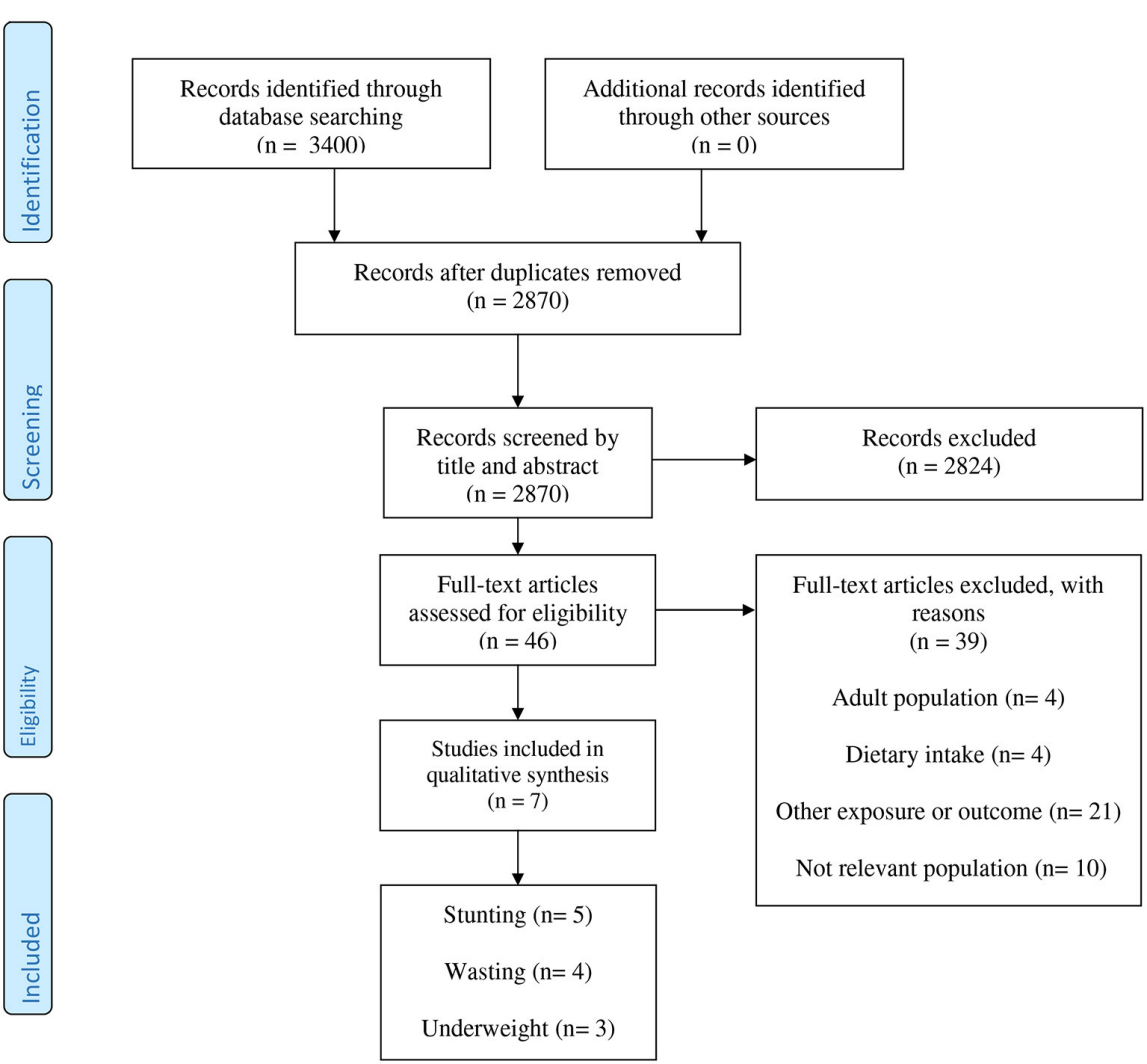
The flow diagram of study selection.

A total of 3,400 records were found by a systematic search that 2,870 remained after removing duplicates (Figure 1). After evaluating essays based on the title and abstract, 2,824 publications were excluded. Among 46 remained publications, 39 papers were excluded for the following reasons: adult population (*n* = 4), dietary intake (*n* = 4), assessing other exposure or outcome (*n* = 21), and not relevant population (*n* = 10).

Finally, seven observational studies (4 cohort and 3 cross-sectional studies) were opted for the systematic review and meta-analysis (14–20). The demographic information of eligible studies has been featured in Table 1. The studies were published between 2015 and 2020 and they were carried out in Tanzania (17, 20), Iran (15, 16), India (18, 19), and Ecuador (14). A total number of 7,624 persons participated in the studies, some of who were stunted (*n* = 1,349), wasted (*n* = 505), and underweight (*n* = 417). Two studies were conducted in infants (5–7 weeks) (17, 20) and five studies were performed in children between 6 months and 9 years old (14–16, 18, 19). Serum vitamin D was assessed by HPLC tandem MS (*n* = 2) (17, 20), and chemiluminescence immunoassay (*n* = 5) (14–16, 18, 19). The studied defined different cut-points for vitamin D deficiency, so that three of them considered serum 25(OH)D <10 ng/ml (15, 16, 18, 20), one considered 25(OH)D <12 ng/ml (19), one considered 25(OH)D <20 ng/ml (17), and other one 25(OH)D <17 ng/ml (20) as vitamin D deficiency. Stunting, wasting, and underweight was defined as a Length-for-age z score (LAZ), weight-for-length z score (WLZ), and weight-for-age z score (WAZ) of 2 or more SDs below the WHO population median, respectively. However, in one study, wasting was defined as BMI-for-age *Z*-score < −1 SD (15), and in another study, stunting considered as a Height-for-age Z-score of < -1 SD (16). All of the included essays had high methodological quality (score ≥ 6) (Supplementary Table 1).

**Table 1:** Characteristics of observational studies eligible in the systematic review and meta-analysis.

**References**	**Country**	**Participants**	**Study design**	**Sample size (gender)**	**Age**	**Follow-up period**	**Outcome**	**Adjustments**	**Quality score**
Sudfeld et al. (20)	Tanzania	HIV-exposed, uninfected infants	Cohort	873 (male and female)	5–7 weeks	18 weeks	Stunting Wasting Underweight	Baseline maternal factors including age, education, marital status, number of prior pregnancies, household assets, food expenditure per person, underweight, anemia, CD4 T-cell count, and use of antiretrovirals during pregnancy, baseline child factors including sex, exclusive breastfeeding, stunting, wasting, low birth weight, anemia, season of 25(OH)D measurement, and randomized treatment regimen.	7
Sudfeld et al. (17)	Tanzania	HIV-unexposed infants	Cohort	510 (male and female)	5–7 weeks	18 weeks	Stunting Wasting Underweight	Maternal age, maternal education, parity, wealth tertile, child sex, randomized regimen, low birthweight (<2,500 g), prematurity (<37 weeks), breastfeeding method at 6 weeks of age, and season of 25(OH)D assessment	7
Mokhtar et al. (14)	Ecuador	children	Cross-sectional	516 (male and female)	6–36 month	–	Stunting	Age and sex.	8
Chowdhury (18)	India	Children	Cohort	323 (male and female)	12–36 month	6 month	Wasting Underweight	Age, sex, breastfeeding status, log transformed annual family income, family structure, mother's years of schooling, father's years of schooling, baseline levels of vitamin B12, folate, anemia status at baseline	6
Nasiri-abadi et al. (15)	Iran	Pre-school children	Cross-sectional	425 (male and female)	5–7 years	–	Wasting	Gender, region, birth interval, supplement use (vitamin A and iron supplements), relevant medical history (diarrhea, respiratory infection, and fever), serum level ferritin, vitamin D and zinc, birth weight	8
Sharif et al. (16)	Iran	Toddlers	Cross-sectional	4,261 (male and female)	10–36 moth	–	Stunting	Sex and residential area (where appropriate), age, family size, first-rank birth, birth interval with previous child, birthweight, history of diseases (diarrhea, respiratory infection, fever, epitaxy and fauvism), supplement use (including vitamin A, vitamin D, iron and zinc supplements), as well as serum levels of retinol, 25(OH)D3 and zinc, where appropriate.	9
Chowdhury et al. (19)	India	Children	Cohort	716 (male and female)	6–30 months	6–9 years	Stunting	Log folate, log soluble transferrin receptor and log homocysteine level, at baseline and the wealth index, paternal occupational status and maternal education at follow-up and intervention group	7

### Meta-Analysis

#### The Association of Serum Vitamin D Status With Risk of Wasting

**Figure 2:**
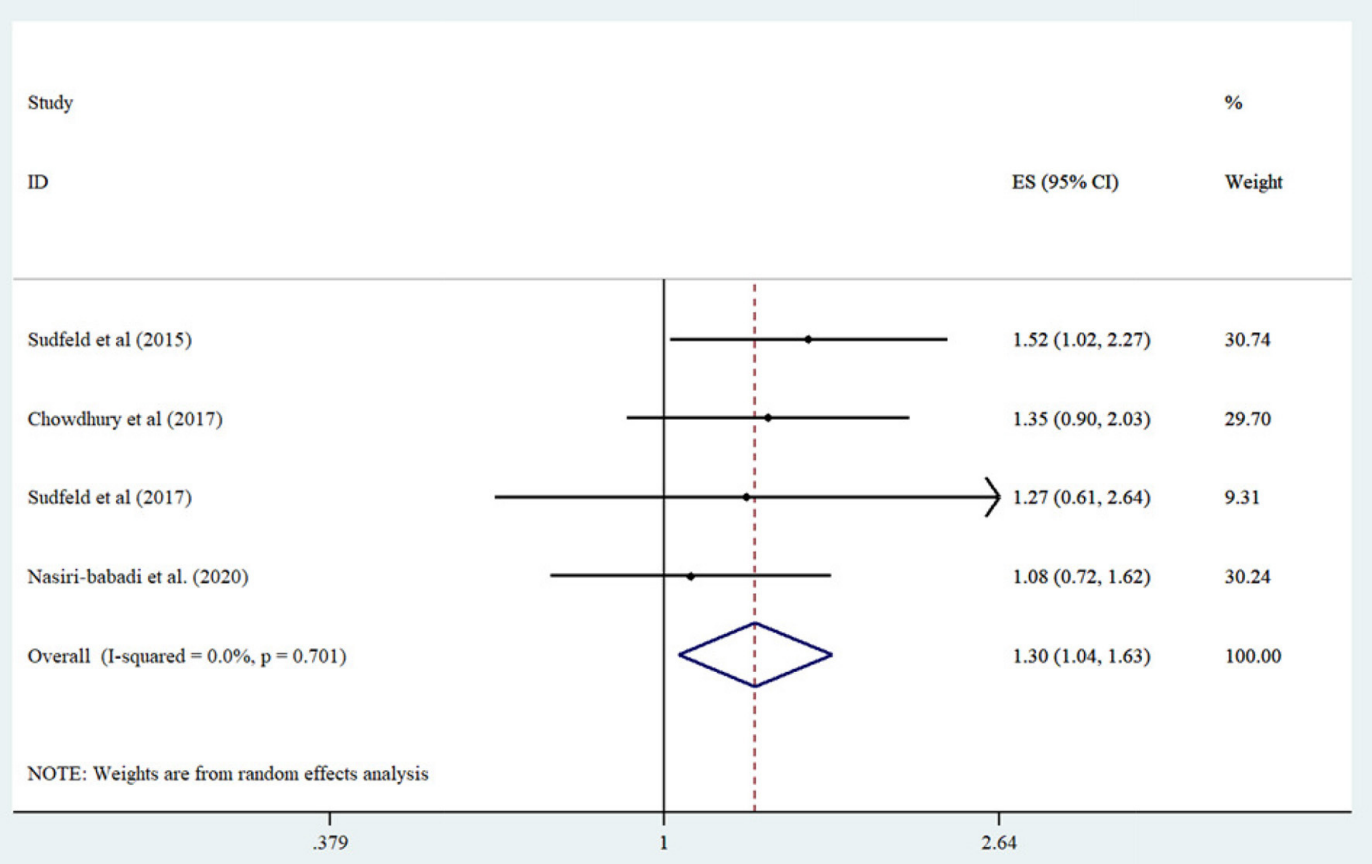
Forest plot derived from random-effects meta-analysis of studies investigating the association between low vs. high serum vitamin D concentration and wasting in children. CI, confidence interval; ES, effect size.

Four studies were included in the analysis of lowest vs. highest serum vitamin D concentrations and risk of wasting (15, 17, 18, 20). Participants with the lowest serum vitamin D concentrations showed a significantly elevated risk of wasting compared to participants in the lowest group (Summary Risk Estimate: 1.30; 95% CI: 1.04, 1.62; *I*^2^ = 0%) (Figure 2). We conducted subgroup analyses based on study design, participant age range, number of cases, and serum vitamin D assessment tool. We also found a significant association between low vs. high serum vitamin D concentrations in cohort studies, those conducted in infants, and studies assessed serum vitamin D by HPLC-MS tool (*P* = 0.036 for all) (Table 2).

**Table 2:** Results of subgroup analysis for vitamin D status and risk of stunting and wasting in children.

	**Number of studies**		**ES (95% CI)**		***P*****-value**		***P*****-heterogeneity**		***I***^****2****^ **(%)**	
	**Stunting**	**Wasting**	**Stunting**	**Wasting**	**Stunting**	**Wasting**	**Stunting**	**Wasting**	**Stunting**	**Wasting**
**Total**	5	4	1.10 (0.72, 1.70)	1.30 (1.04, 1.62)	0.637	0.021	<0.001	0.701	81.6	0
**Study design**										
Cohort	3	3	0.99 (0.75, 1.30)	1.41 (1.08, 1.84)	0.948	0.012	0.599	0.880	0	0
Cross-sectional	2	1	0.88 (0.72, 1.06)	1.08 (0.72, 1.62)	0.180	0.710	<0.001	–	95	–
**Participants**										
Infants	2	2	0.92 (0.66, 1.29)	1.45 (1.02, 2.07)	0.657	0.036	0.599	0.673	0	0
Children	3	2	0.90 (0.76, 1.08)	1.20 (0.90, 1.60)	0.288	0.202	<0.001	0.448	95	0
**Number of cases^a^**										
<300	3	2	0.99 (0.75, 1.30)	1.33 (0.93, 1.90)	0.948	0.117	0.227	0.886	26.4	0
≥300	2	2	0.88 (0.72, 1.06)	1.28 (0.96, 1.70)	0.180	0.087	<0.001	0.241	81.3	27.3
**Assessment of vitamin D**										
HPLC-MC assay	2	2	0.92 (0.66, 1.29)	1.45 (1.02, 2.07)	0.657	0.036	0.456	0.673	0	0
Chemiluminescence immunoassay	3	2	0.90 (0.76, 1.08)	1.20 (0.90, 1.60)	0.288	0.202	<0.001	0.448	90.5	0

a *<100, ≥100 for wasting*.

*CI, Confidence Interval; ES, Effect Size; HPLC-MC, high-performance liquid chromatography-tandem mass spectrometry*.

#### The Association of Serum Vitamin D Status With Stunting Risk

**Figure 3:**
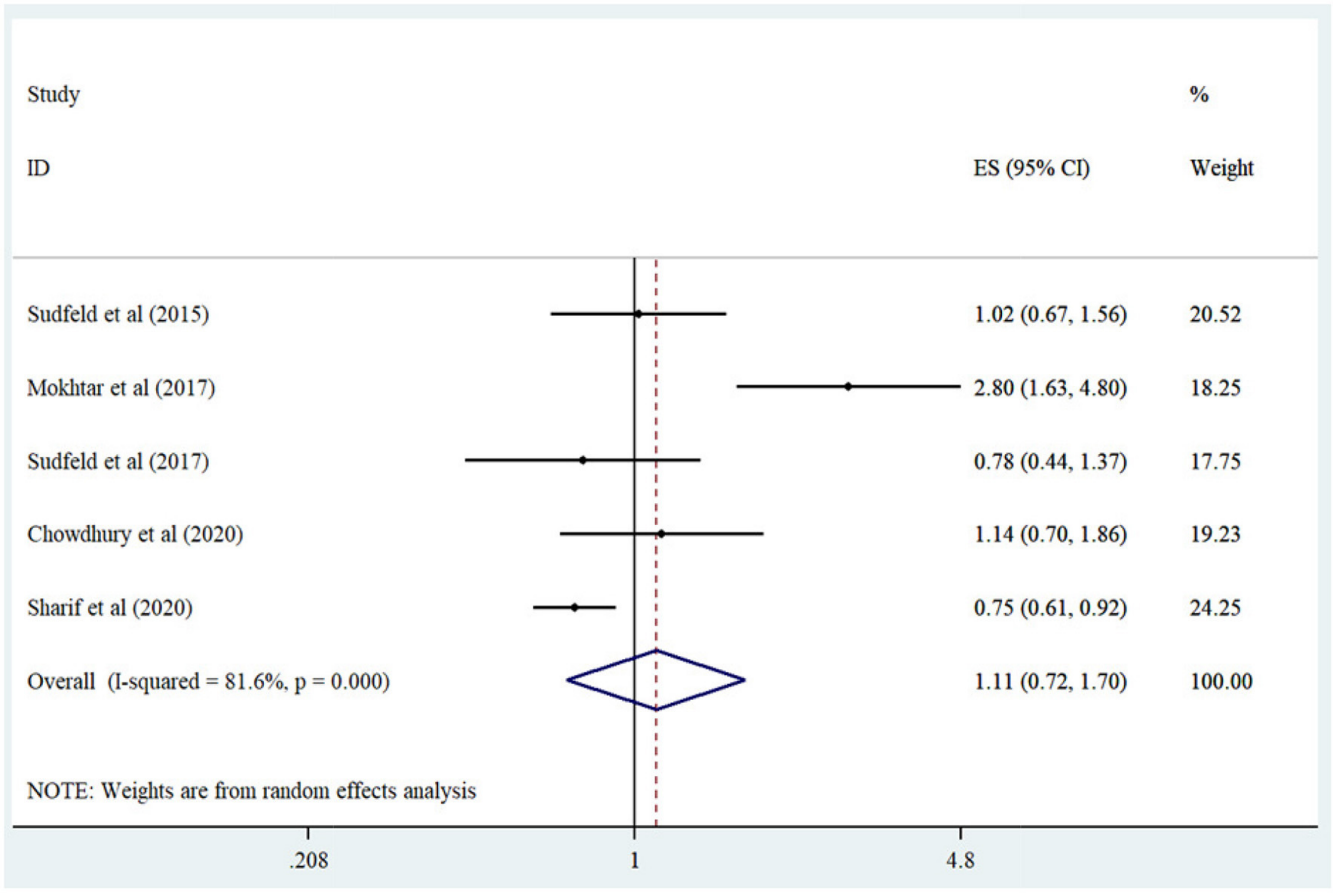
Forest plot derived from random-effects meta-analysis of studies investigating the association between low vs. high serum vitamin D concentration and stunting in children. CI, confidence interval; ES, effect size.

Five studies were pooled in the meta-analysis comparing high vs. low serum level vitamin D categories (14, 16, 17, 19, 20). Comparing the lowest vs. highest serum vitamin D concentrations, no significant association was demonstrated between serum vitamin D level and stunting risk (Summary Risk Estimate: 1.10; 95% CI: 0.72, 1.70; *I*^2^ = 81.6%) (Figure 3). Subgroup analysis based on predefined factors including study design, participant age range, number of cases, and serum vitamin D assessment tool displayed that all of these items were the sources of heterogeneity (Table 2). Moreover, we failed to find any significant relationship between serum vitamin D status and stunting in subgroups.

#### The Association of Serum Vitamin D Status With Risk of Underweight

**Figure 4:**
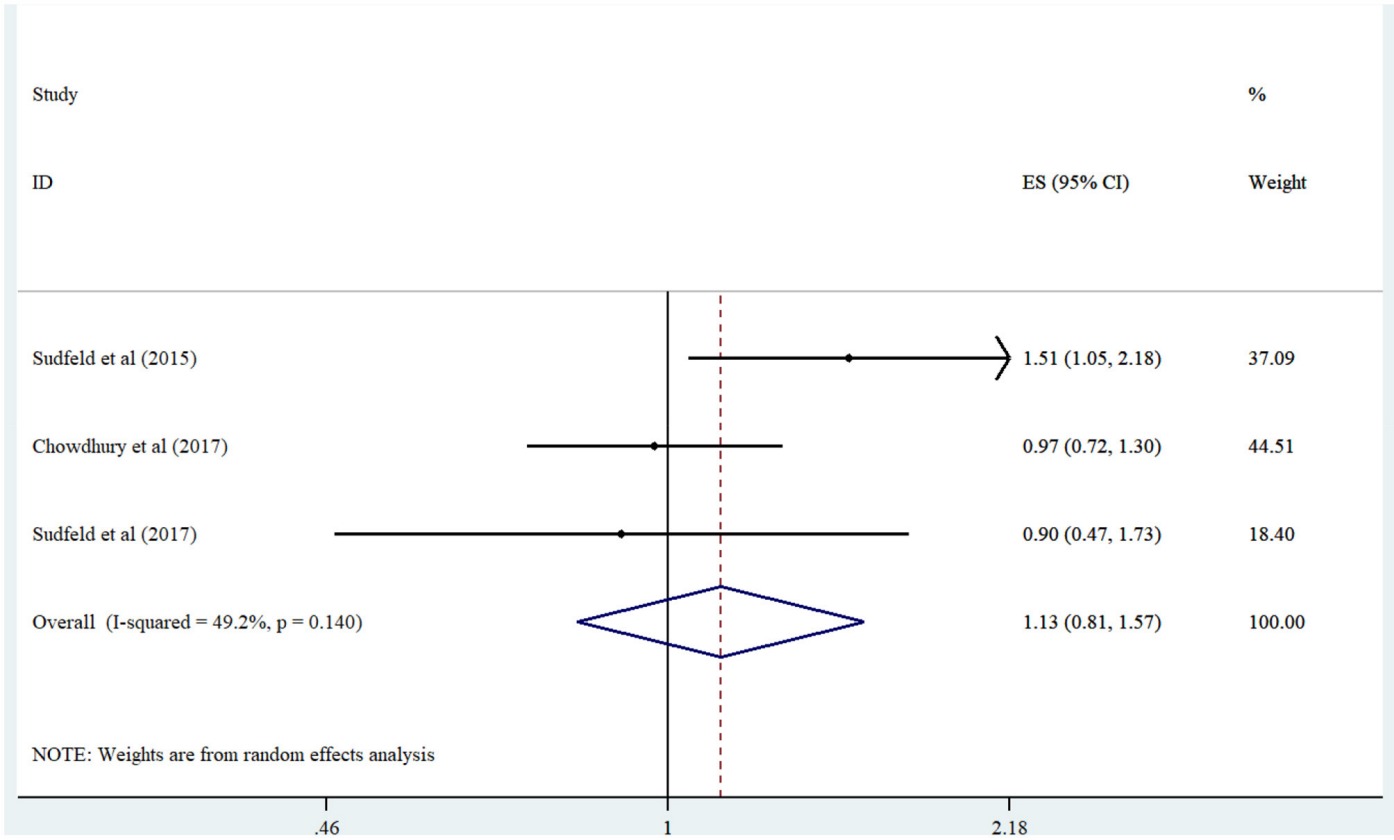
Forest plot derived from random-effects meta-analysis of studies investigating the association between low vs. high serum vitamin D concentration and underweight in children. CI, confidence interval; ES, effect size.

Combining the risk estimates of three studies (17, 18, 20) regarding the association of lowest vs. highest serum vitamin D concentration and risk of underweight illustrated no significant relationship (Summary Risk Estimate: 1.12; 95% CI: 0.81, 1.56; *I*^2^ = 49.2%) (Figure 4).

### Publication Bias

Evidence of publication bias was not recognized in both the egger test and funnel plot for wasting (*P* = 0.93), stunting (*P* = 0.20), and underweight (*P* = 0.97) (Supplementary Figures 1–3).

### Sensitivity Analysis

According to the sensitivity analysis test results, any studies could not significantly influence summary risk regarding the association of low vs. high serum concentrations of vitamin D and risk of wasting, stunting, and underweight (Supplementary Figures 4–6).

## Discussion

The present meta-analysis assessed the association between vitamin D status and risk of wasting, stunting, and underweight using data from three cohort and four cross-sectional studies. Our findings demonstrated a significant association between lowest vs. highest serum vitamin D concentration and risk of wasting. However, serum vitamin D status (low vs. high) was not associated with an increased risk of stunting and underweight.

We found significant inverse relationship between vitamin serum vitamin D and wasting. In line with our result, a randomized clinical trial study in Pakistan showed supplementation with 200,000 IU twice/month vitamin D3 improved the mean WLZ in children aged 6–58 months with severe acute malnutrition (25). Another clinical trial on Canadian healthy mature infants who used at least 400 IU/d vitamin D supplement from 1 to 12 months of age found that elevated vitamin D concentrations from infancy to 3 years of age were related to leaner body composition (26). In contrast to our finding, a cross-sectional study in Uganda on 158 children aged 6–24 months observed no significant difference in vitamin D levels between the wasting and non-wasting children (27).

The chances are that differences in study design, age group, assessment method of serum vitamin D concentration, number of wasted children, defining different cut-points for vitamin D deficiency, and adjusted factors are the reasons for these contradictions. In this study, we also found similar findings in cohort studies, infants, studies with a higher number of cases, and studies that used HPLC-MS to evaluate vitamin D concentration. Needing follow-up assessments to examine the associations between vitamin- D status and growth, the findings of prospective cohort studies are more reliable than cross-sectional studies. Moreover, infants are more susceptible to vitamin D deficiency than children in higher ages due to low vitamin D intake during breastfeeding, confined exposure to sunlight, and inadequate vitamin D transmission through the placenta (28). Furthermore, 25(OH)D concentrations can be overestimated by the immunoassay method compared to HPLC-MS (29) due to its lipophilic nature, which makes it vulnerable to matrix effects in the protein binding assays (30).

We detected that risk of stunting and underweight did not elevated in low serum vitamin D. In line with our finding, a double-blind, placebo-controlled trial on 3,046 children ages 1–11 months from inner-city Kabul indicated that supplementation with 100,000 IU vitamin D3 for 18 months had no effect on weight age, weight for height, height for age (31). In contrast to our findings, Kumar et al., in a randomized clinical trial, investigated the effects of 35 μg/week vitamin D in low birth weight infants (1.8–2.5 kg) from birth up to 6 months of age in India. They indicated that vitamin D supplements raised weight, height, and arm circumference compared to placebo (32). Besides, maternal vitamin D3 supplementation (35,000 IU/wk) during the third trimester of pregnancy improved infants' linear growth during 1 year after birth compared to infants born from mothers who did not receive vitamin D supplementation (33).

Therefore, the lack of significant relationships between vitamin D status and stunting and underweight in our study may be unpredicted. One likelihood is that bone growth disorders in children occur after a severe vitamin D deficiency. Another explanation is that in addition to vitamin D shortage, other growth-related macro and micronutrient deficiencies such as animal-source protein, calcium, vitamin B12, and zinc may also involve low growth (34). Thus, the vitamin D impact on development could be trivial in the light of other growth-related nutrient deficiencies in children.

Vitamin-D plays several roles in the growth of children (14, 18). First, children need higher calcium intake than adults to provide adequate calcium for bone mineralization (35). Vitamin D is participated in bone mineralization by maintaining normal calcium and phosphate concentrations in the blood (36). Second, Vitamin D increases the growth plate cells' sensitivity to growth hormone, which has an important influence on linear growth in school-aged children (9). Third, vitamin D may help muscle growth through vitamin D receptors (VDR) (37). It conducts this role by stimulation of the production of new proteins involving in muscle cell proliferation, differentiation, and contractility and the regulation of calcium transport in the sarcoplasmic reticulum (38, 39).

Based on our knowledge, this study is the first systematic review and meta-analysis to investigate the association of vitamin D status with the risk of wasting, stunting, and underweight in children. Moreover, we did not identify any evidence of publication bias. The amount of heterogeneity was low regarding the association of vitamin D status with the risk of wasting and underweight. Finally, all of included essays had high quality score.

This study has several limitations. First, the small number of studies and sample size may have led to the disability to identify some significant associations. Second, although all included studies controlled different types of relevant confounders, it may be necessary to consider other residual confounding factors, including dietary and serum levels of other growth-limited nutrients, in future studies. Third, using cross-sectional studies may prevent us from detecting causal associations between vitamin D status and undernutrition factors. Finally, the number of studies that provided adequate data for dose-response analysis was so low that we could not perform this analysis.

In conclusion, we found that children with the lowest serum vitamin D concentration had a greater risk of wasting than children with the highest serum vitamin D level category. However, there was no significant relationship between serum vitamin D status and risk of stunting and underweight in children. More prospective cohort studies with larger sample size and longer duration of follow-up among various children populations, including those living in areas with a higher prevalence of vitamin D deficiency like tropical settings, are needed to confirm our findings.

These study report important public health implications particularly for populations with low socioeconomic status. Our findings propose that strategies to enhance vitamin D status in children by food fortification or supplementation could help the Ministry of Health's efforts to decrease undernutrition especially wasting.

## Data Availability Statement

The original contributions presented in the study are included in the article/Supplementary Material, further inquiries can be directed to the corresponding author/s.

## Author Contributions

ChunhS, HL, and HS designed the work. BW and ChunlS extracted the data. ChunlS analyzed the data. HL wrote the manuscript and supervised the work. All authors critically revised and approved the final version of the manuscript.

## Conflict of Interest

The authors declare that the research was conducted in the absence of any commercial or financial relationships that could be construed as a potential conflict of interest.
