# Supplementary material for: Association Between Vitamin D Status and Undernutrition Indices in Children: A Systematic Review and Meta-Analysis of Observational Studies
Source: Front Pediatr. 2021 Jun 4;9:665749. doi: 10.3389/fped.2021.665749 (PMC8211725; doi:10.3389/fped.2021.665749)
Supplement: Supplementary file 1 [file Data_Sheet_1.docx]

**Supplementary Table:** Quality assessment of included studies based on Newcastle-Ottawa Scale

| **Code** | **First author (year)** | **Selection** | **Comparability** | **Outcome** | **Overall** |
| --- | --- | --- | --- | --- | --- |
| 1 | Sudfeld, C. R.et al. (2015) | *** | ** | ** | 7 |
| 2 | Sudfeld, C. R.et al. (2017) | *** | ** | ** | 7 |
| 3 | Mokhtar, R. R et al (2017) | **** | * | *** | 8 |
| 4 | Chowdhury, R., et al. (2017) | ** | ** | ** | 6 |
| 5 | Nasiri-Babadi, P. et al. (2020) | *** | ** | *** | 8 |
| 6 | Sharif, Y, et al. (2020) | **** | ** | *** | 9 |
| 7 | Chowdhury, R., et al. (2020) | ** | ** | *** | 7 |

**Supplementary Figure 1**


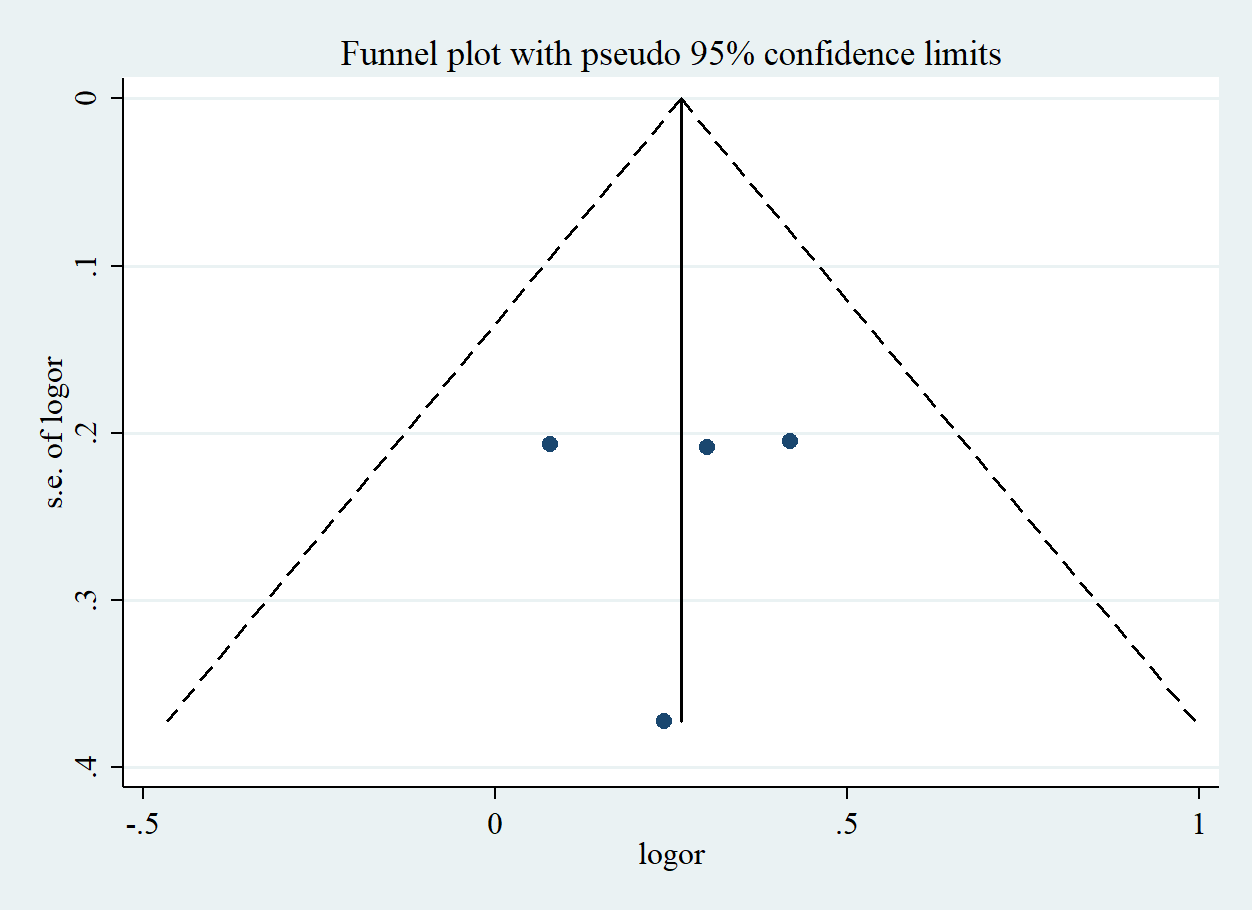


Funnel plot of the risk estimates of four studies on the association between lowest versus highest serum vitamin D level and risk of wasting in children. Each dot represents one study. Egger’s test P=0.93. SE, standard error.

**Supplementary Figure 2**


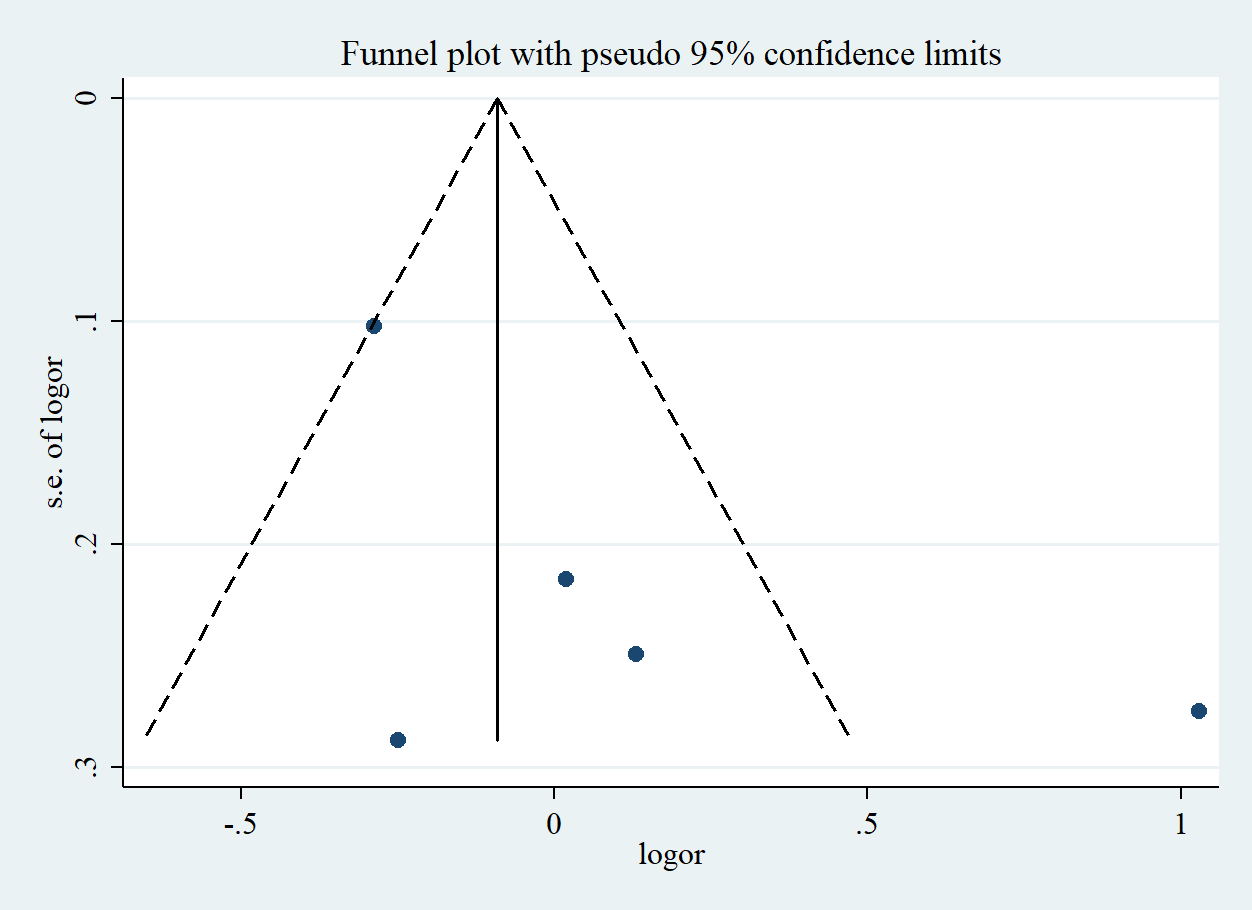


Funnel plot of the risk estimates of five studies on the association between lowest versus highest serum vitamin D level and risk of stunting in children. Each dot represents one study. Egger’s test P=0.20. SE, standard error.

**Supplementary Figure 3**

**
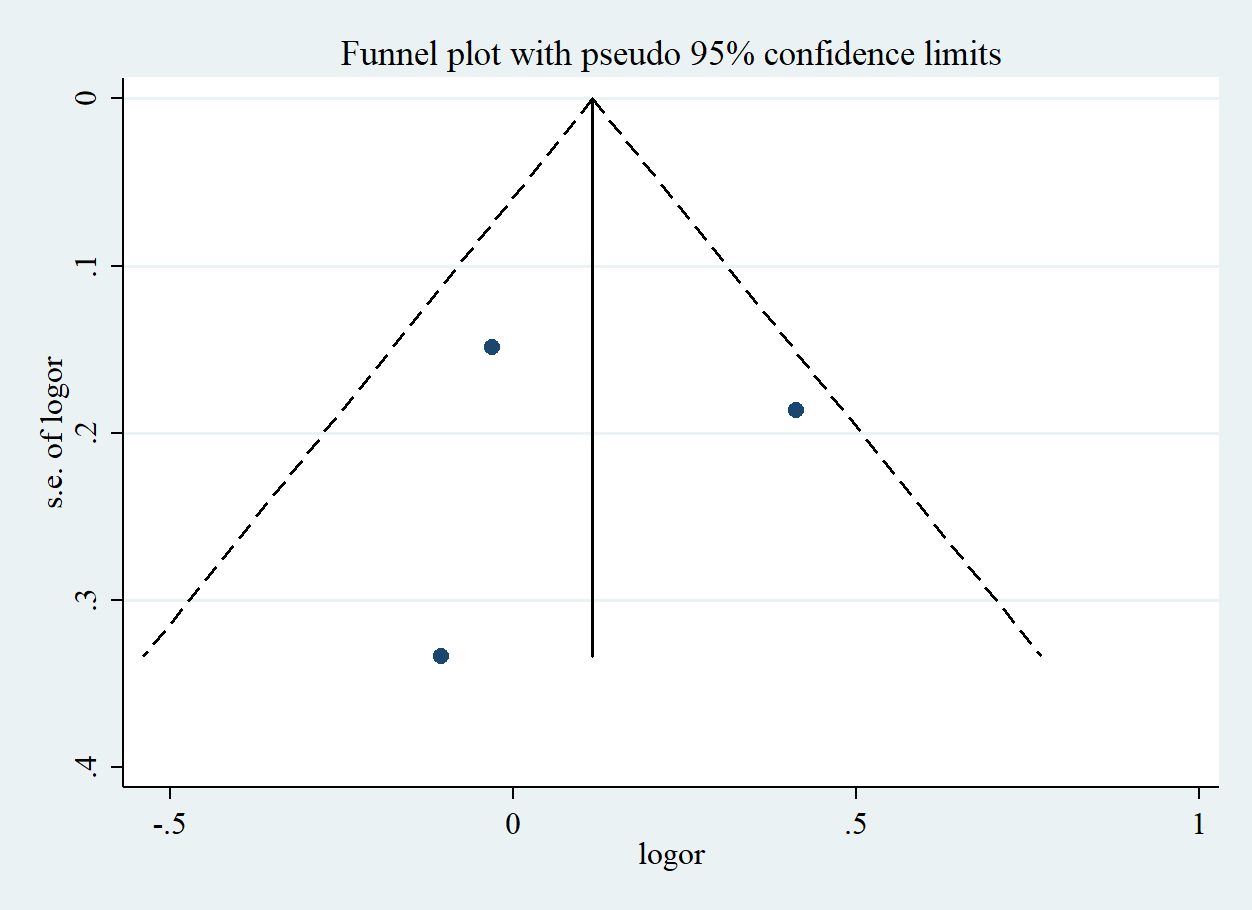
**

Funnel plot of the risk estimates of five studies on the association between lowest versus highest serum vitamin D level and risk of underweight in children. Each dot represents one study. Egger’s test P=0.97. SE, standard error.

**Supplementary Figure 4**

Sensitivity analysis of the association between lowest versus highest serum vitamin D level and risk of wasting in children. CI, confidence interval

**Supplementary Figure 5**

Sensitivity analysis of the association between lowest versus highest serum vitamin D level and risk of stunting in children. CI, confidence interval

**Supplementary Figure 6**

Sensitivity analysis of the association between lowest versus highest serum vitamin D level and risk of underweight in children. CI, confidence interval
